# Tracing Functional Antigen-Specific CCR6^+^ Th17 Cells after Vaccination

**DOI:** 10.1371/journal.pone.0002951

**Published:** 2008-08-13

**Authors:** Johann Pötzl, Catherine Botteron, Eugen Tausch, Xiomara Pedré, André M. Mueller, Daniela N. Männel, Anja Lechner

**Affiliations:** 1 Institute of Immunology, University of Regensburg, Regensburg, Germany; 2 Department of Neurology, University of Regensburg, Regensburg, Germany; 3 Multiple Sclerosis Research Center of New York, New York, New York, United States of America; New York University School of Medicine, United States of America

## Abstract

**Background:**

The function of T helper cell subsets in vivo depends on their location, and one hallmark of T cell differentiation is the sequential regulation of migration-inducing chemokine receptor expression. CC-chemokine receptor 6 (CCR6) is a trait of tissue-homing effector T cells and has recently been described as a receptor on T helper type 17 (Th17) cells. Th17 cells are associated with autoimmunity and the defence against certain infections. Although, the polarization of Th cells into Th17 cells has been studied extensively in vitro, the development of those cells during the physiological immune response is still elusive.

**Methodology/Principal Findings:**

We analysed the development and functionality of Th17 cells in immune-competent mice during an ongoing immune response. In naïve and vaccinated animals CCR6^+^ Th cells produce IL-17. The robust homeostatic proliferation and the presence of activation markers on CCR6^+^ Th cells indicate their activated status. Vaccination induces antigen-specific CCR6^+^ Th17 cells that respond to in vitro re-stimulation with cytokine production and proliferation. Furthermore, depletion of CCR6^+^ Th cells from donor leukocytes prevents recipients from severe disease in experimental autoimmune encephalomyelitis, a model for multiple sclerosis in mice.

**Conclusions/Significance:**

In conclusion, we defined CCR6 as a specific marker for functional antigen-specific Th17 cells during the immune response. Since IL-17 production reaches the highest levels during the immediate early phase of the immune response and the activation of Th17 cells precedes the Th1 cell differentiation we tent to speculate that this particular Th cell subset may represent a first line effector Th cell subpopulation. Interference with the activation of this Th cell subtype provides an interesting strategy to prevent autoimmunity as well as to establish protective immunity against infections.

## Introduction

In the late 80ies Mossman et al. designated Th1 and Th2 cells according to their capacity to secrete the cytokines IFN-γ and IL-4, respectively [1]. Since then, IL-10 and TGF-β producing T cells were identified as regulatory T cells [2]. The increasing knowledge about the heterogeneity of the Th cell subpopulations helped to explain how most infections are resolved. Nevertheless, the involvement of Th cells in the elimination of extracellular bacteria and fungi, and in the development of autoimmune disorders could not be adequately explained with the T effector cell subpopulations as defined in the literature so far. Recent studies on the role of the APC-derived cytokine IL-23 for the development of autoimmunity and clearance of infections [3]–[6] expanded our knowledge in T cell immunology by pointing to a new Th cell subtype involved in these mechanisms. These particular Th cells are characterised by the production of the cytokine IL-17 and are now referred to as Th17 cells [7], [8]. While the expansion and maintenance of Th17 cells depends on IL-23 [9], their generation in mice is induced by the early inflammatory cytokines IL-21, IL-6, and TGF-β [9]–[11]. These cytokines are produced early in the inflammatory process by innate effector cells and Th cells [12], [13]. Once the Th cells are differentiated, the cytokine milieu influences the functionality of each of the Th cell subsets; Thl, Th2, and Th17 cells produce cross-regulatory cytokines that are mutually inhibitory for the respective other subsets [8], [14]. Nevertheless, it is still unclear how the development and maintenance of Th17 cells is regulated during the immune response in vivo. One of the reasons for this is the lack of well-characterised surface markers identifying Th17 cells. Based on the findings that Th17 cells express CCR6 [15], [16] we examined the generation of IL-17 producing Th cells in a non-infectious vaccination model with ovalbumin (OVA), and analysed the functionality of Th17 cells during the immune response in immune-competent C57BL/6 mice. For the first time, we demonstrated that CCR6 is a selective marker for the identification of Th17 cells during an immune response. Furthermore, CCR6^+^ Th17 cells were present even in naïve mice and this population showed signs of activation in the steady state. During the acute phase of the immune response, the proportion of IL-17 producing Th cells in the lymph nodes decreased compared to the Th1 cell subpopulation. Nevertheless, antigen-specific IL-17 producing responder cells arose in the lymph node. The functionality of CCR6^+^ Th17 cells was demonstrated in adoptively transferred experimental autoimmune encephalomyelitis (at-EAE); transfer of lymph node cells containing CCR6^+^ Th17 cells led to severe disease while depletion of CCR6^+^ Th cells attenuated the course of at-EAE.

## Materials and Methods

### Mice

Inbred C57BL/6 mice were purchased from Charles River (Sulzfeld, Germany), inbred SJL/J mice were obtained from Harlan Winkelmann (Borchen, Germany). OVA TCR transgenic (OT-II) mice were bred and maintained in the animal facilities of the University of Regensburg. Mice of 8–16 weeks of age were used. Vaccination with OVA was performed injecting 100 µg OVA and 8 nmol CpG-ODN 1668 [17] s.c. into the hind footpad. All experiments were conducted according to animal experimental ethics committee guidelines and were approved by the local authorities.

### Antibodies and reagents

The following antibodies were used for flow cytometry: Rat anti-mouse CD4 (clone RM4-5), rat anti-mouse CD8α (clone 53-6.7), rat anti-mouse CD44 (clone IM7), rat anti-mouse CD62L (clone MEL-14), hamster anti-mouse CD69 (clone H1.2F3), rat anti-mouse IFN-γ (clone XMG1.2), rat anti-mouse IL-17 (clone TC11-18H10.1), mouse anti-BrdU (clone B44), PE and APC labelled isotype control IgG. All mAb and isotype controls were purchased from BD Biosciences (Heidelberg, Germany). Rat anti-mouse CCR6 (clone 140706) was purchased from R&D Systems (Wiesbaden, Germany). Hamster anti-mouse CD3ε (clone 145.2C11) was purified from hybridoma cell culture supernatant using protein-G-sepharose columns (GE Healthcare, München, Germany).

### Flow cytometry and cell sorting

Popliteal and inguinal lymph nodes were harvested. Single cell suspension was prepared as previously described [18]. Cells were incubated with the appropriate antibodies in staining buffer (PBS containing 2% FCS). Data was collected on the LSR II flow cytometer (BD Biosciences) and analysed using the DIVA software (BD Biosciences). All staining profiles were based on live-gated cells, as determined by forward and sideward scatter properties. For the separation of CD4^+^ T cells the MACS system (Miltenyi Biotec, Bergisch Gladbach, Germany) was employed using anti-mouse CD4 MACS beads. For further separation of CD4^+^ CCR6^+^ lymphocytes 5×10^7^ cells per sample were incubated in 500 µl of staining buffer with the appropriate antibodies. Cells were sorted on the FACSAria cell sorter (BD Biosciences).

### Analysis of cell proliferation in vitro

Lymph node cells (5×10^5^) were labelled with CFSE (Invitrogen, Karlsruhe, Germany) at a final concentration of 1.25 µM, and cultured in 200 µl RPMI 1640 (PAN Biotech, Aidenbach, Germany) supplemented with 10% FCS (Sigma-Aldrich, München, Germany), 50 µM β-mercaptoethanol, 2 mM L-glutamine, 100 U/ml penicillin and 100 µg/ml streptomycin (PAN Biotech). Cells were stimulated with OVA (20 µg/ml; Sigma-Aldrich) or plate bound anti-CD3 (5 µg/ml) and proliferation was assessed by flow cytometry.

### Measurement of cell proliferation in vivo

OT-II mice were immunised for the indicated periods of time and fed with 5-Bromo-2′-deoxyuridine (BrdU; 800 µg/ml) in drinking water for three days. Draining lymph nodes were harvested and single cell suspensions were prepared. Cell surface staining was performed. The detection of the incorporated BrdU was conducted with the BrdU Flow Kit (BD Biosciences) according to the manufacturer's instructions with the exception that a FITC-conjugated mouse anti-BrdU (clone B44) was used for detection of the thymidine analogue. Proliferation of either CD4^+^ CCR6^+^ or CD4^+^ CCR6^−^ cells was assessed by the presence of BrdU fluorescence in dividing cells.

### Determination of cytokine production

Cells were stimulated with plate bound anti-CD3 (5 µg/ml) or OVA (20 µg/ml) in vitro and supernatants were collected. IFN-γ and IL-17A protein quantification was performed with DUO-ELISA kits (R&D Systems) following the manufacturer's instructions. The detection limit for IFN-γ and for IL-17A was 20 pg/ml.

### Intracellular cytokine staining

Freshly isolated or in vitro reactivated lymph node cells were stimulated with ionomycin (1 µM; Sigma-Aldrich), and PMA (80 ng/ml; Sigma-Aldrich) in the presence of GolgiStop (BD Biosciences) for 4 h. Intracellular staining was performed using the Cytofix/Cytoperm Kit (BD Biosciences) according to the manufacturer's instructions.

### Induction and assessment of at-EAE

SJL/J mice were immunised by injection of 200 µg of murine PLP (aa139–151, Pepceuticals Limited, UK), emulsified in Complete Freund's adjuvant (CFA) (Difco Laboratories, Detroit, Michigan, USA) supplemented with 500 µg Mycobacterium tuberculosis (Difco Laboratories). After 10 days cells were harvested from draining lymph nodes and single cell suspension was prepared. Lymphocytes were sorted into CD4^−^, CD4^+^ CCR6^−^ and CD4^+^ CCR6^+^ fractions. After the sorting procedure cell fractions either including or excluding CD4^+^ CCR6^+^ cells were combined in the same ratio as present in the starting lymph nodes and cultured with 5 µg/ml PLP for 72 h. Afterwards, lymph node cells were harvested and washed twice with PBS. Each recipient mouse received 3.5×10^6^ activated lymph node cells by i.p. injection. Weight and clinical score were recorded at the indicated time points according to the following criteria: 0 = healthy; 1 = limp tail; 2 = partial hindlimb weakness and/or ataxia; 3 = complete paralysis of at least one hindlimb; 4 = severe forelimb weakness; 5 = moribund or dead.

### Statistical analysis

Error bars represent SEM. For statistical analysis two-tailed Student's t test was used unless indicated otherwise; p<0.05 was considered significant. For at-EAE experiments differences were analyzed by the non-parametric Mann-Whitney U test; p<0.05 was considered significant.

## Results

### Analysis of CCR6^+^ Th cells in immune-competent C57BL/6 mice

Antigen-specific T cell response occurs in distinct steps. After activation Th cells differentiate into effector cells that migrate to their target organs, and orchestrate the immune response. Therefore, re-organization of adhesion molecules and chemokine receptors is a hallmark of T cell differentiation [19].

**Figure 1:**
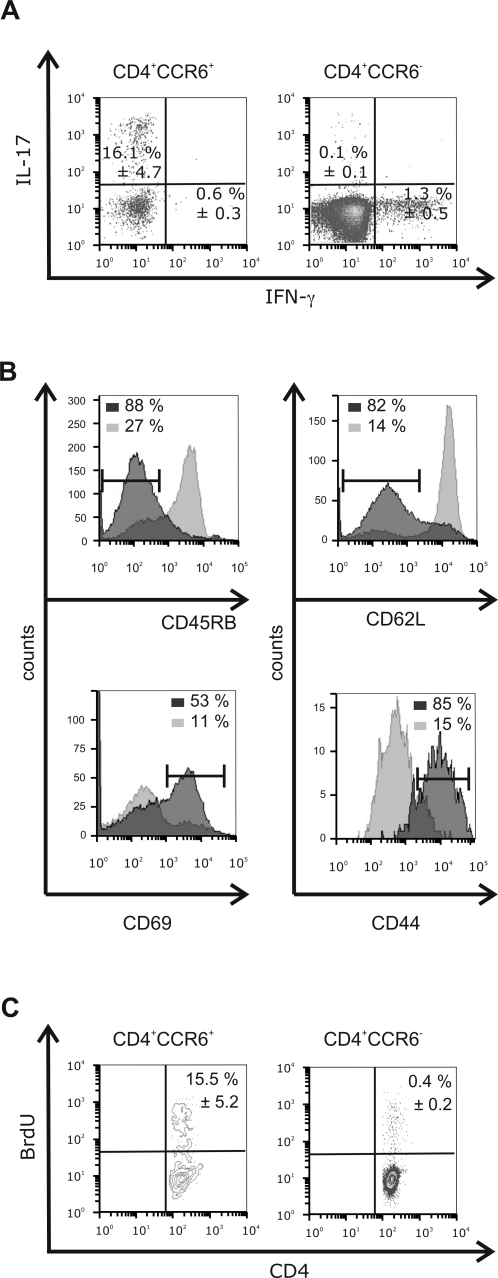
Residual CD4^+^ CCR6^+^ T cells show an activated phenotype in C57BL/6 mice. (A and B) Lymph nodes of naïve C57BL/6 mice were harvested and cells were analysed by multicolour flow cytometry. For each experiment lymph node cells from three mice were pooled. (A) Cells were gated on CD4^+^ cells and the expression of CCR6 and IL-17 or CCR6 and IFN-γ was monitored. The percentage of cytokine producing cells in the live cell gate was determined. Data is given as mean (±SEM) of three independent experiments. (B) The expression of activation markers on CD4^+^ CCR6^+^ (dark grey histogram) and CD4^+^ CCR6^−^ (light grey histogram) cells was analysed. Data is representative for three independent experiments. (C) OT-II mice were fed with BrdU. Cells were isolated from lymph nodes. In vivo proliferation of Th cells was assessed by their BrdU-linked fluorescence. Representative density plots of gated CD4^+^CCR6^+^ (left panel) and CD4^+^CCR6^−^ (right panel) cells are shown. Data is given as mean (±SEM) of six independent experiments.

In this study we analysed the fate and the function of CCR6^+^ Th cells in naïve and vaccinated mice. Analysis of the cytokine pattern of lymph node residing CCR6^+^ Th cell fraction in naïve mice showed that this T cell subpopulation clearly consists of Th17 cells; 16.1% (±4.7) of the CD4^+^ CCR6^+^ Th cell fraction produced IL-17 (Fig. 1A, left panel). In the respective Th cell fraction nearly no IFN-γ production was observed (0.6%±0.3; Fig. 1A, left panel). In addition, neither IL-4 nor the anti-inflammatory cytokine IL-10 were detectable in CCR6^+^ Th cells (data not shown). Th cells that were negative for CCR6 did not produce IL-17 (Fig. 1A, right panel). The exclusive production of the cytokine IL-17 allowed us to follow the generation of Th17 cells according to the surface marker CCR6 during the immune response in our study.

Since the CCR6^+^ IL-17 producing cells were present in naïve mice we were interested in the phenotype of those cells. CCR6^+^ Th cells expressed the activation markers CD69 and CD44 (Fig. 1B) in untreated mice. In contrast to the former markers, CD62L and CD45RB are characteristics of naïve Th cells and were down regulated on CCR6^+^ Th cells (Fig. 1B). The expression pattern of the activation markers and adhesion molecules on CCR6^+^ Th cells pointed to an activated phenotype of these cells in naïve mice; it did not change substantially after immunisation (data not shown). To correlate the activated phenotype with functional properties on CCR6^+^ Th cells we determined in vivo proliferation of those cells in non-vaccinated C57BL/6 mice. In naïve mice the CCR6^+^ Th cell subpopulation proliferated vigorously. 15.5% (±5.2; Fig. 1C) of the CCR6^+^ Th cells incorporated BrdU within the treatment period of three days. Nearly no proliferation was detected in CCR6^−^ Th cells (0.4% cells in proliferation±0.2; Fig. 1C).

### Vaccination favours the development of IFN-γ producing Th1 cells

**Figure 2:**
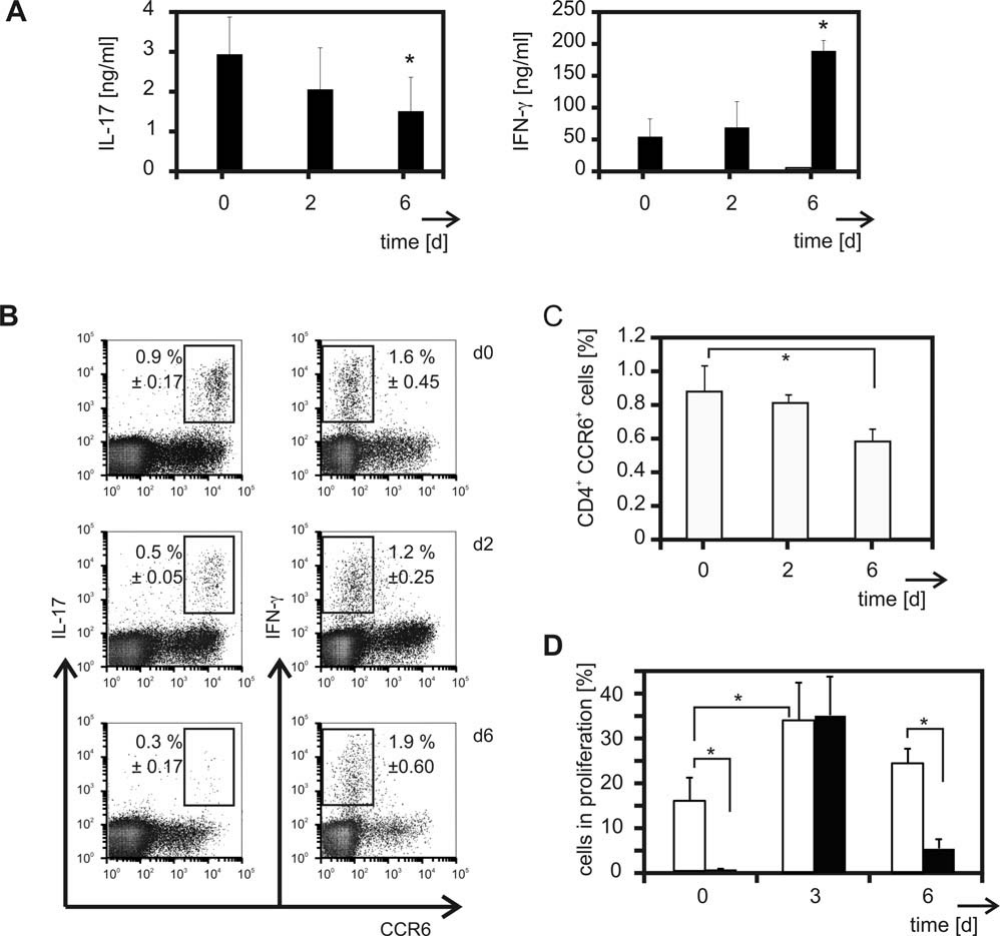
Production of IL-17 and IFN-γ is differentially regulated during the immune response. (A–C) C57BL/6 mice were immunised for the indicated periods of time. For each experiment lymph node cells from three mice were pooled. (A) Cells were cultured in the presence of plate bound anti-CD3. After 72 h in culture supernatants were collected. The amounts of IL-17 (left panel) and IFN-γ (right panel) were analysed by ELISA. ELISA was performed in duplicates. Data is given as mean (±SEM) of two independent experiments, * p<0.05. (B) Intracellular cytokine staining was performed immediately after isolation as described in materials and methods. The percentage of IL-17 and IFN-γ producing cells (±SEM) within the CD4^+^ T cell fraction was determined by flow cytometry. Three independent experiments were performed. (C) CD4^+^ lymphocytes were analysed for the expression of CCR6. Mean values (±SEM) of three independent experiments are shown, * p<0.05. (D) OT-II mice were immunised for the indicated periods of time. In vivo proliferation of CD4^+^ CCR6^+^ (open bars) and CD4^+^ CCR6^−^ (black bars) cells was determined according to their BrdU-linked fluorescence. Data is given as mean values (±SEM) of three (day 6) to six (day 0 and d 3) independent experiments. * p<0.01.

To clarify the role of CCR6^+^ Th17 cells during the immune response we vaccinated C57BL/6 mice with OVA and CpG-oligodeoxynucleotides (ODN) as adjuvant. These conditions mimic the situation during infections with pathogens. During the acute phase of the T cell response on day six after vaccination lymph node cells had a reduced capacity to produce IL-17 protein after polyclonal activation (Fig. 2A left panel). As in naïve animals IL-17 production was restricted to the CCR6^+^ Th cell subpopulation after immunisation (Fig. 2B, left panel). Following immunisation IFN-γ became the dominant cytokine produced by lymph node cells (Fig. 2A, right panel). Thereby, IFN-γ was synthesised by CCR6^−^ Th cells (Fig. 2B, right panel) and cytotoxic T lymphocytes (CTL; data not shown). To rule out whether these findings are due to the use of CpG-ODN we performed additional immunisation experiments using alum and IFA as adjuvants. Both alum precipitated OVA and OVA emulsified in IFA induced the same reduction in Th17 cells during the peak phase of the immune response as seen in CpG-OVA vaccinated mice (Fig. S1). The decrease in IL-17 producing Th cells after vaccination correlated with the reduction of CCR6^+^ Th cells in the lymph nodes (Fig. 2C). To elucidate the mechanisms underlying the different kinetics of the induction of IL-17 and IFN-γ, respectively, we determined the kinetics of the expansion of CCR6^+^ and CCR6^−^ cells during the course of the immune response in vivo. Immunisation led to a tremendous induction of proliferation of the CCR6^−^ Th cell subpopulation (Fig. 2D; 34.7%±8.6). Between days three and six of the immune reaction the expansion of CCR6^−^ cells was almost completed and the proliferation rate dropped to 5.4% of proliferating cells (±2.1). As CCR6^+^ Th cells showed a high proliferation rate in naïve mice (Fig. 1C and 2D), the responsiveness of CCR6^+^ Th cells to vaccination was rather weak but clearly detectable (Fig. 2D; 33.6%±8.4).

### Antigen-specific CCR6^+^ Th17 cells are generated in response to OVA in C57BL/6 mice

**Figure 3:**
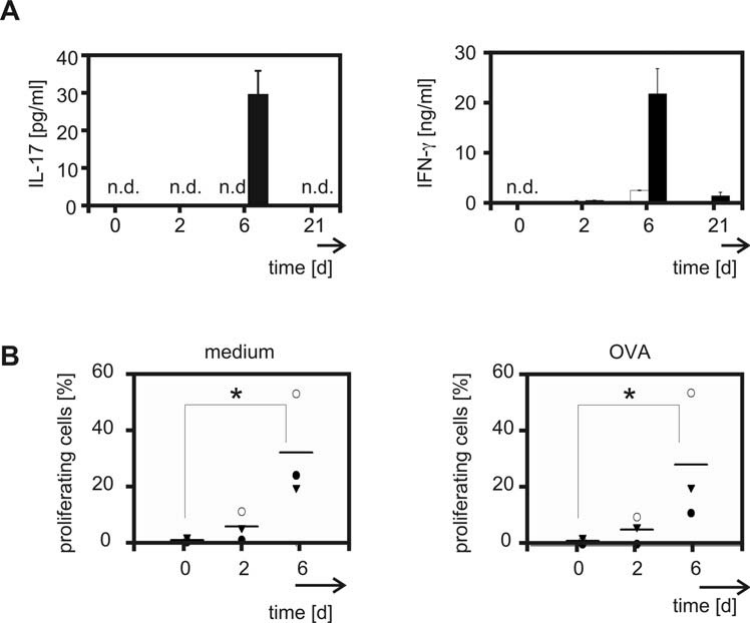
Induction of antigen-specific CCR6^+^ Th17 cells after vaccination. C57BL/6 mice were immunised for the indicated periods of time. Lymph node cells were isolated and stimulated in vitro. (A) OVA-stimulated (black bars) or control cells (open bars) were cultured for 72 h in vitro. The amounts of IL-17 and IFN-γ were quantified. ELISA was performed in duplicates. Mean values (±SEM) of three independent experiments are depicted (n.d. indicates values below the detection limit). (B) Lymph node cells of three mice were pooled, labelled with CFSE and cultured for 96 h. Proliferation of OVA-stimulated (right panel) and control CD4^+^ CCR6^+^ cells (left panel) was determined by CFSE dilution. Each data point represents one experiment (n = 3/time point). Horizontal bars indicate the mean. * p<0.05.

So far we noticed proliferation of CCR6^+^ Th cells following immunisation with OVA and CpG-ODN but the antigen-specificity of these vaccination-induced cells still needed to be clarified. Therefore, we tested whether in vivo activated Th cells are capable to produce IL-17 in response to in vitro re-stimulation with specific antigen. Cells obtained either from naïve mice or mice that were immunised for two days did not release IL-17 in response to OVA-challenge. On day six after immunisation cells produced considerable amounts of IL-17 (Fig. 3A, left panel). In accordance to polyclonal activation antigen-specific re-stimulation resulted in a strong induction of IFN-γ release (Fig. 3A, right panel).

**Figure 4:**
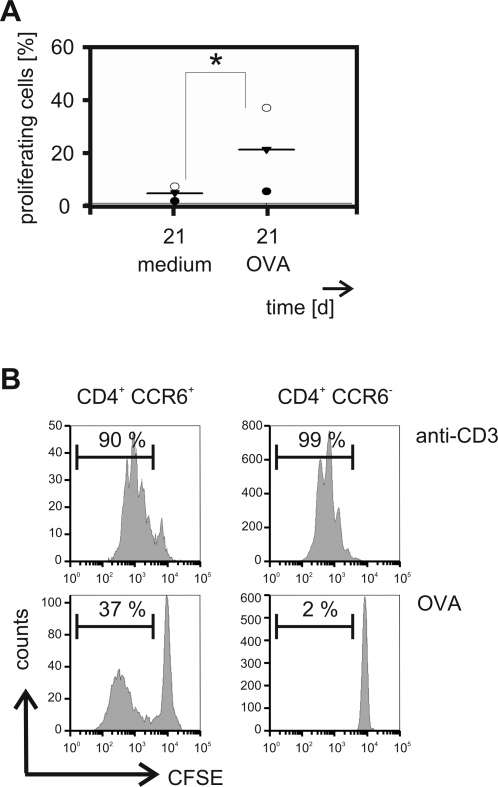
On day 21 after immunisation CD4^+^ CCR6^+^ proliferated in response to specific antigen. C57BL/6 mice were immunised for 21 days. Lymph nodes cells of three mice were pooled for each experiment and labelled with CFSE. (A) Cells were cultured for 96 h in the absence or presence of OVA. Proliferation was measured by flow cytometry. The proportion of proliferating cells within the CD4^+^ CCR6^+^ fraction was determined. Each data point represents one experiment (n = 3/time point). Horizontal bars indicate the mean. * p<0.05. (B) Cells were stimulated with anti-CD3 or OVA and proliferation was assessed by flow cytometry. The percentage of proliferating cells is depicted. One representative experiment out of three is show.

We next determined the capacity of in vivo activated CCR6^+^ Th17 cells to expand in vitro. On day six after vaccination we detected antigen-induced proliferation of the CCR6^+^ Th cell subset (Fig. 3B, right panel). Cells cultured without exogenous antigen also progressed in divisions (Fig. 3B, left panel). This suggests that endogenous antigen derived from the previous vaccination induced proliferation in those cells. This hypothesis was supported by the fact that T cells obtained from mice on day 21 after immunisation did not proliferate without re-stimulation but responded to the administration of OVA (Fig. 4A). Moreover, at that late point of the immune response antigen-specific reactivity was largely limited to the CCR6^+^ Th cell subpopulation. Whereas CCR6^+^ and CCR6^−^ Th cells proliferated to the same extent upon polyclonal stimulation only a small proportion of the CCR6^−^ Th cell subpopulation showed responsiveness to in vitro challenge with antigen (Fig. 4B).

### CCR6 defines functional antigen-specific Th17 cells that induce autoimmune disease

**Figure 5:**
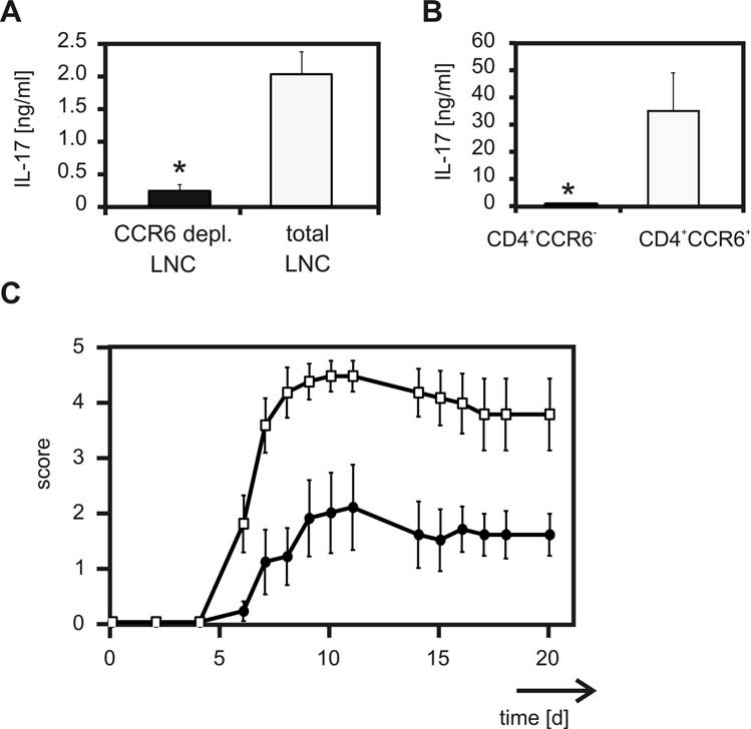
Depletion of CD4^+^ CCR6^+^ T cells attenuates disease development in at-EAE. SJL/J mice were immunised as described in materials and methods. On day 10 lymph nodes were harvested and cells were isolated. (A) Total lymph node cells (open bar) or lymph node cells depleted of CD4^+^ CCR6^+^ cells (black bar) were cultured in vitro in the presence of PLP for 72 h. The production of IL-17 protein was quantified. ELISA was performed in duplicates. Data represents the mean values (±SEM) of two independent experiments. * p<0.05. (B) Purified CD4^+^ CCR6^+^ (open bar) or CD4^+^ CCR6^−^ (black bar) T cells were stimulated with plate bound anti-CD3 for 72 h. IL-17 was quantified. ELISA was performed in duplicates. Data represents the mean values (±SEM) of three independent experiments. * p<0.05. (C) Mean EAE scores (±SEM) from mice received either total lymph node cells (open squares) or CD4^+^ CCR6^+^ depleted lymph node cells (closed circles) are shown (n = 5/group). The differences between the two groups were statistically significant by day 6 through day 17. * p<0.05.

To investigate whether CCR6 could be used as a specific marker to isolate functional Th17 cells we induced EAE in mice. In this murine model for multiple sclerosis the autoimmune deterioration of the central nervous system (CNS) is mainly mediated by inflammatory Th17 cells [20]. Antigen-specific Th cells are primed by vaccination with the myelin-specific antigen PLP. Disease is induced transferring in vivo activated lymph node cells into naïve recipient mice. Therefore, depletion of CCR6^+^ Th cells in that particular transfer model should prevent disease development in mice. In accordance with our previous observations we observed a significant reduction of IL-17 production in PLP re-stimulated lymph node cells depleted of CCR6^+^ Th cells (Fig. 5A). Analysis of sorted CCR6^+^ and CCR6^−^ Th cell fractions revealed that IL-17 was exclusively produced by CCR6^+^ Th cells (Fig. 5B). Although, lymph node cells that were depleted for CD4^+^CCR6^+^ cells regained CCR6 expression (Fig. S2A) nearly no IL-17 production was detected in those cells after in vitro re-stimulation for 3 days (Fig. S2B). Consequently, adoptive transfer of lymph node cells containing CCR6^+^ Th cells into naïve recipients induced severe EAE whereas depletion of CCR6^+^ Th cells form donor cells considerably attenuated the severity of disease in recipient mice, and reduced their mortality (Fig. 5C and Table 1).

**Table 1:** Depletion of CD4^+^ CCR6^+^ T cells attenuates disease in the at-EAE model.

Transferred cell type	Incidence a	Mortality a	Mean day of disease onset	Maximum score	Cumulative score^b^
Total LNC^c^	5/5	3/5	6 (±0; n = 5)	4.5 (±0.7; n = 5)	46.7 (±14.1; n = 5)
CD4^+^CCR6^+^ depleted LNC^c^	4/5	0/5	10 (±4.6; n = 5)^d^	2.6 (±1.6; n = 5)^d^	18.1 (±13.9; n = 5)^d^

a Clinical outcome of EAE of mice that had received either total lymph node cells or CD4^+^CCR6^+^ depleted lymph node cells is shown for the observation period of 20 days.

b Cumulative score was calculated by summing up each individual score registered during the follow-up period.

c LNC, lymph node cells

d p<0.05 compared with control mice; Mann-Whitney U test.

## Discussion

Differential expression of chemokine receptors is an accepted principle for the classification of different Th cell subpopulations [21], [22]. The chemokine receptor CCR6 is expressed on activated Th cells but the function of CCR6^+^ Th cells has been rather obscure for a long time [23]. Recent reports on Th17 cells start to enlighten the role of this particular T cell subset [15]. In our study we demonstrated for the first time that CCR6 is a valid marker to define functional antigen-specific Th17 cells in mice. In naïve and immunised mice IL-17 producing Th cells from lymph nodes exclusively resided within the CCR6^+^ Th cell fraction. Analysis of the cytokine production of sorted CCR6^+^ Th cells clearly showed that this Th cell fraction was the only source of IL-17 protein within the Th cell population.

A common issue linking CCR6 expressing Th cells and Th17 cells is their association with autoimmune diseases [24]–[26]. IL-17 producing Th cells have been shown to be responsible for the development of EAE in mice [20]. As a matter of fact depletion of auto-reactive Th17 cells on the basis of CCR6 expression from lymph node cells attenuated the encephalitogenicity of transferred cells in at-EAE. It is important to note that IL-17 producing CTL [20] could not compensate for the loss in IL-17 producing Th cells resulting from the depletion of the CCR6^+^ Th cell fraction. The ablation of CCR6^+^ Th cells from lymph node cells of PLP immunised mice almost completely prevented IL-17 production. This experiment places emphasis on the correlation between IL-17 production and CCR6 expression. Interestingly, in vitro re-stimulation of primed lymph node cells that were depleted for CD4^+^CCR6^+^ cells, led to the emergence of CCR6^+^ T cells but not Th17 cells. As we were not able to completely block EAE development by deletion of CCR6^+^ Th17 cells, it is conceivable that those cells regain functionality in vivo irrespective of their inability to produce IL-17 during in vitro culture.

Up to now the generation of murine Th17 cells is accomplished by polarizing these cells in vitro in the presence of cytokines such as IL-6 and TGF-β [9], [11]. Additionally, the spontaneous development of CCR6^+^ Th17 cells could be shown in autoimmune prone mice [27] but the stimulatory requirements for the development of Th17 cells in the course of an infection are yet ill-defined. In vitro IL-17 production can be forced by stimulating lymph node cells with pathogenic components, such as LPS, CpG DNA motifs, and pertussis toxin [9], [28]. Those toll-like-receptor-activating substances trigger the production of pro-inflammatory cytokines by antigen presenting cells that further induce Th cell differentiation towards the Th17 subtype. Thus, our non-infectious vaccination scheme provides the opportunity to follow the formation of Th17 cells in vivo.

In fact, we were able to induce antigen-specific Th17 cells by our protocol. These cells were functional by producing IL-17 protein and expanding in response to their specific antigen without the presence of additional polarizing cytokines. The presence of IL-17 in antigen-specifically re-stimulated cell cultures suggested the differentiation of Th17 cells from non-committed naïve T cells after vaccination. However, since Th17 cells are most abundant in naïve mice we cannot completely rule out whether that cytokine is derived from pre-existing CCR6^+^ memory cells. This implies the presence of cross-reactive T cells within the IL-17 producing Th cell subpopulation. Polyspecific T cells are part of the normal T cell repertoire and initially selected against self- or microbial antigens [29], [30]. Although, the promiscuity in antigen recognition is thought to be in particular important for T cell memory and homeostasis it often leads to autoimmunity [31]–[34]. Indeed, IL-17 production in our experiments is accompanied with Th cells with a memory phenotype. Since those cells are resistant to suppression by regulatory T (Treg) cells [35] the occurrence of polyspecificity within the Th17 cell subset might explain the detrimental role of Th17 cells for the development of chronic inflammatory diseases.

Strikingly, our data demonstrate that Th1 cells became the predominant Th cell subtype during the peak of the physiological inflammatory response. As IFN-γ negatively regulates IL-17 production in vitro [8] this cytokine also seems to display a suppressive effect on Th17 cells in vaccinated mice. Since we observed a reduction of Th17 cells under Th1, and Th2 polarizing conditions these observations might reflect the differential temporary needs for Th1 or Th2 cells and Th17 cells during the course of an infection, and account for the different kinetics underlying the development of these Th cell subtypes. Th17 cells are equipped with homing receptors that allow them to reside in peripheral tissues. We also noticed a robust proliferation and expression of activation-induced receptors within the CCR6^+^ Th cell subset in non-vaccinated mice. This pointed to a Th cell subpopulation that is constantly on the alert. Th cell-derived IL-17 induces the production of growth factors, cytokines, and chemokines by stromal cells [36], [37]. Thus, Th17 cells provide the stimulatory conditions creating the appropriate environment needed for the recruitment of innate immune cells early in inflammation [38], [39]. Subsequently, during the peak of the immune response Th1 cells assume their role in promoting the functionality of CTL and macrophages.

An open question still remains: is ligation of CCR6 required for in vivo activation of Th17 cells? Since blocking CNS-expressed CCL20, the cognate ligand of CCR6, in active EAE results in an attenuation of the disease [40], [41] CCR6 expression seems to be necessary for the generation of functional Th17 cells. Accordingly, CCR6^−/−^ mice exhibit impaired Th cell function in delayed type of hypersensitivity reactions (DTH) and in re-infection experiments [18], [42]. These inflammatory conditions also test primed Th cells for their functionality. One likely explanation is that in those models the CCR6-mediated migration of Th cells into the target organs is needed for a sufficient activation of Th17 cells. In contrast, in the respective adoptive transfer EAE model blocking CCL20 did not prevent the development of disease [40]. That indicates once activated CCR6^+^ Th17 cells remain functional. They react against endogenous antigen and cause autoimmunity. Together, these findings point to a defect in the differentiation towards the Th17 subtype in the absence of CCR6.

From our observations, we conclude that CCR6^+^ Th cells represent an activated Th cell subpopulation. They are capable of producing IL-17 after vaccination with protein antigens. However, exceeded stimulation of Th17 cells results in a continuous production of IL-17 that promotes autoimmune phenomena [43]. Therefore, Th17 cells have to be controlled effectively. CCR6 is a common attribute of certain tissue homing Treg cells [44] and Th17 cells, and the dichotomy between Treg cells and inflammation-promoting Th17 cells has been stated recently [11]. Equipped with a similar type of homing and activation receptors both Th cell subtypes might meet at the sites of inflammation and cross-regulate each other. Extending the knowledge of the mechanisms influencing the generation and recruitment of Th17 cells in vivo, and the identification of additional target structures on these cells may lead to new therapeutic approaches for the treatment of autoimmune disorders or to the development of innovative vaccination strategies.

## Supporting Information

Figure S1 IL-17 production is down regulated during the immune response. (A–C) C57BL/6 mice were immunised with 100 µg OVA either adsorbed to Imject® alum (Pierce Biotechnology, Rockford, USA) or emulsified in incomplete Freund's adjuvant (IFA). Draining lymph nodes of naïve mice (black bar) and mice immunised with Ova alum (grey bars) or IFA (open bars) were harvested at the indicated time points. (A) Cells were stimulated in vitro with plate bound anti-CD3 for 72 h. The production of IL-17 protein was quantified. ELISA was performed in duplicates. * p<0.05; compared to day 0. (B) Cells were applied for intracellular cytokine staining immediately after isolation. The percentage of IL-17 producing CCR6^+^ cells (±SEM) within the CD4^+^ T cell fraction was determined by flow cytometry. * p<0.05; compared to day 0. (C) Intracellular cytokine staining of lymph node cells obtained on day 6 after immunisation was performed as described in materials and methods. The percentage of IL-17 producing CCR6^+^ cells within the CD4^+^ T cell fraction was determined by flow cytometry. Data represents the mean values±SEM (n = 3, control mice; n = 4 immunised mice).
(0.55 MB TIF)

Figure S2 CCR6 expression does not concur with IL-17 production in PLP re-stimulated lymph node cells. SJL/J mice were immunised as described in materials and methods. (A) Total lymph node cells (open bar) or lymph node cells depleted of CD4^+^ CCR6^+^ cells (black bar) were re-stimulated with PLP for 72 h in vitro. The percentage of CCR6^+^ cells within the CD4^+^ T cell fraction is shown. Data represents the mean values±SEM (n = 4). (B) After the 72 h re-stimulation period intracellular cytokine staining was performed as described in materials and methods. Cells were gated on CD4^+^ CCR6^+^ cells and the percentage of IL-17 producing cells was calculated. Data is given as mean±SEM (n = 4; * p<0.05).
(0.21 MB TIF)
