# Supplementary figures and images for: Tracing Functional Antigen-Specific CCR6+ Th17 Cells after Vaccination
Source: PLoS One. 2008 Aug 13;3(8):e2951. doi: 10.1371/journal.pone.0002951 (PMC2491584; doi:10.1371/journal.pone.0002951)

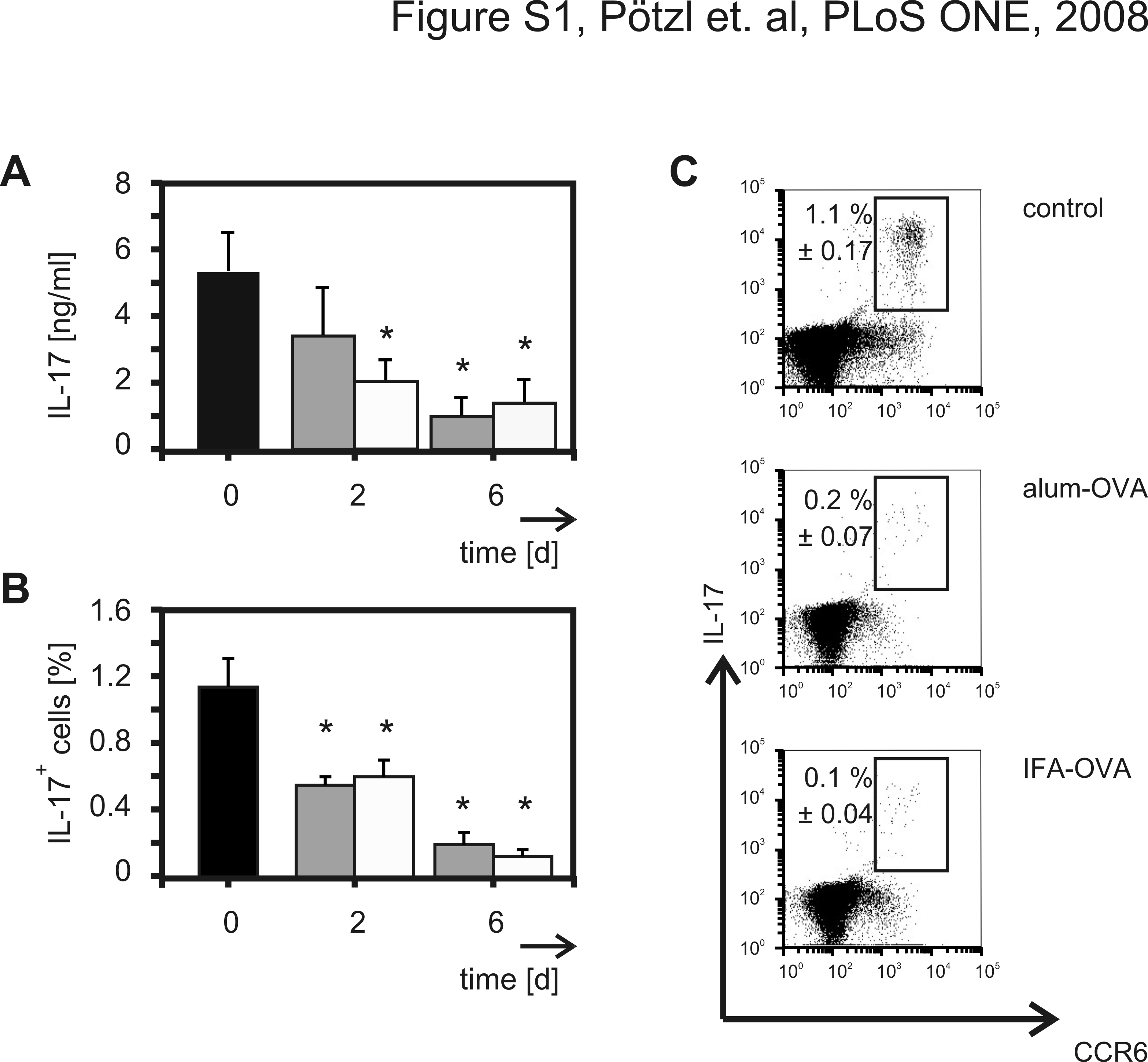

Supplement: Figure S1 — IL-17 production is down regulated during the immune response. (A–C) C57BL/6 mice were immunised with 100 µg OVA either adsorbed to Imject® alum (Pierce Biotechnology, Rockford, USA) or emulsified in incomplete Freund's adjuvant (IFA). Draining lymph nodes of naïve mice (black bar) and mice immunised with Ova alum (grey bars) or IFA (open bars) were harvested at the indicated time points. (A) Cells were stimulated in vitro with plate bound anti-CD3 for 72 h. The production of IL-17 protein was quantified. ELISA was performed in duplicates. * p<0.05; compared to day 0. (B) Cells were applied for intracellular cytokine staining immediately after isolation. The percentage of IL-17 producing CCR6+ cells (±SEM) within the CD4+ T cell fraction was determined by flow cytometry. * p<0.05; compared to day 0. (C) Intracellular cytokine staining of lymph node cells obtained on day 6 after immunisation was performed as described in materials and methods. The percentage of IL-17 producing CCR6+ cells within the CD4+ T cell fraction was determined by flow cytometry. Data represents the mean values±SEM (n = 3, control mice; n = 4 immunised mice). (0.55 MB TIF) [file pone.0002951.s001.tif]

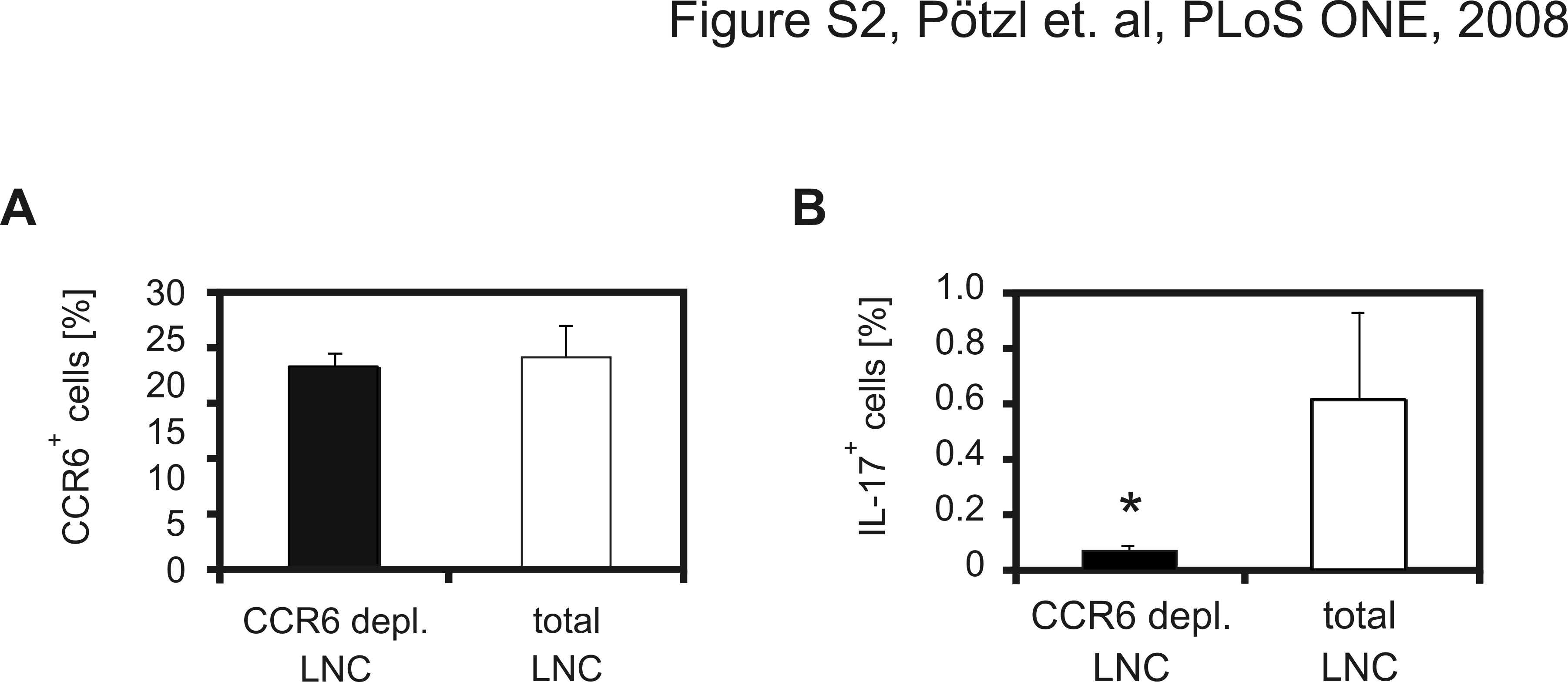

Supplement: Figure S2 — CCR6 expression does not concur with IL-17 production in PLP re-stimulated lymph node cells. SJL/J mice were immunised as described in materials and methods. (A) Total lymph node cells (open bar) or lymph node cells depleted of CD4+ CCR6+ cells (black bar) were re-stimulated with PLP for 72 h in vitro. The percentage of CCR6+ cells within the CD4+ T cell fraction is shown. Data represents the mean values±SEM (n = 4). (B) After the 72 h re-stimulation period intracellular cytokine staining was performed as described in materials and methods. Cells were gated on CD4+ CCR6+ cells and the percentage of IL-17 producing cells was calculated. Data is given as mean±SEM (n = 4; * p<0.05). (0.21 MB TIF) [file pone.0002951.s002.tif]
